# Contact-tracing in cultural evolution: a Bayesian mixture model to detect geographic areas of language contact

**DOI:** 10.1098/rsif.2020.1031

**Published:** 2021-08-11

**Authors:** Peter Ranacher, Nico Neureiter, Rik van Gijn, Barbara Sonnenhauser, Anastasia Escher, Robert Weibel, Pieter Muysken, Balthasar Bickel

**Affiliations:** ^1^ University Research Priority Program (URPP) Language and Space, University of Zurich, Zurich, Switzerland; ^2^ Department of Geography, University of Zurich, Zurich, Switzerland; ^3^ Center for the Interdisciplinary Study of Language Evolution (ISLE), University of Zurich, Zurich, Switzerland; ^4^ Department of Slavonic Languages and Literatures, University of Zurich, Zurich, Switzerland; ^5^ Department of Comparative Language Science, University of Zurich, Zurich, Switzerland; ^6^ Leiden University Centre for Linguistics, Leiden, Netherlands; ^7^ Centre for Language Studies, Radboud University Nijmegen, Nijmegen, Netherlands

**Keywords:** Bayesian clustering, cultural evolution, linguistic areas, spatial analysis, confounding, language, mixture model

## Abstract

When speakers of different languages interact, they are likely to influence each other: contact leaves traces in the linguistic record, which in turn can reveal geographical areas of past human interaction and migration. However, other factors may contribute to similarities between languages. Inheritance from a shared ancestral language and universal preference for a linguistic property may both overshadow contact signals. How can we find geographical contact areas in language data, while accounting for the confounding effects of inheritance and universal preference? We present sBayes, an algorithm for Bayesian clustering in the presence of confounding effects. The algorithm learns which similarities are better explained by confounders, and which are due to contact effects. Contact areas are free to take any shape or size, but an explicit geographical prior ensures their spatial coherence. We test sBayes on simulated data and apply it in two case studies to reveal language contact in South America and the Balkans. Our results are supported by findings from previous studies. While we focus on detecting language contact, the method can also be used to uncover other traces of shared history in cultural evolution, and more generally, to reveal latent spatial clusters in the presence of confounders.

## 1. Introduction

Speaker communities are rarely, if ever, completely isolated from each other. Communication between different communities requires finding a common language. This may lead to situations of bi- or multilingualism. Exposure to another language, especially if this is widespread within a community and takes place over a long period of time, can lead to horizontal transfer: the incorporation of words or structural features from one language into another. Although the importance of language contact for understanding the evolution of languages was acknowledged already in the 19th century [1], modelling its effects remains a challenge in language data and in patterns of cultural evolution more generally [2–9].

Contact effects can take many shapes and sizes and can be the result of a number of distinct processes. The most readily recognizable effects involve borrowing of forms (and functions) from one language to another. Commonly, this involves the borrowing of lexicon (e.g. English borrowed the word *language* from French) but may also involve structural material, such as affixes or individual sounds (e.g. suffixes like *-able*, as in *readable*, are borrowed from French).

When these types of contact effects spread from one language to another, it may lead languages spoken in a more or less contiguous area to become similar in their properties. The resulting areas of linguistic convergence are generally referred to as a linguistic area or *Sprachbund*. An example is the linguistic area of western and central Europe where languages tend to share several properties more commonly than in the adjacent regions of Asia, e.g. a system of definite and indefinite articles (English ‘the’ versus ‘a’, Spanish ‘el/la’ versus ‘un(a)’, Hungarian ‘a(z)’ versus ‘egy’) [10]. Detecting such areas is challenging and problem-ridden [2,3,11–13], as they are the result of a number of complex historical processes that are difficult to reconstruct. How can we find geographical areas where languages have been in contact using empirical data and statistical inference?

A straightforward way of answering this question would be to look for shared features between geographically proximate languages. However, inferring contact from this alone ignores two important confounding effects that can also contribute to similarities between languages: inheritance and universal preference.

— Inheritance: Languages are transmitted from one generation to the next in an evolutionary process akin to the descent with modification that characterizes biological evolution [14,15]. In language, the modification stems from variation that each generation adds, mostly for signalling social identities. While this can lead to the split of a language into dialects and eventually into new languages, many properties persist and are inherited faithfully. As a result, languages may share a property just because they split from the same ancestral language and the property survived the split (or indeed several splits). An example is the inheritance of gender distinctions in many Indo-European languages (e.g. Italian, Russian and Hindi).
— Universal preference: The structure of languages is shaped by universal aspects of how they are used for communication and thought, how they are processed in the brain and how they are expressed with our speech and gesture systems. As a result, languages may share a property just because all languages tend to have it [16–20]. An example is the observation that virtually all languages have a formal means to distinguish questions from statements (e.g. intonation or a special word), with only very few exceptions [21].

Contact effects have generally been considered to be those (non-chance) similarities that are neither due to inheritance nor to universal preference. However, it is exceedingly difficult to attribute similarities categorically to contact, inheritance, or universal developments because the relevant processes interact in complex ways [2]. For example, a property that is universally preferred is also likely to be inherited when languages split and to be borrowed in contact. Or, when languages are in contact over many generations, it is likely that they all tend to inherit the same properties. What is needed, therefore, is a probabilistic way of estimating the relative contribution of each process.

In statistical terms, the task of finding contact areas can be described as clustering, i.e. finding groups of objects whose members share commonalities. However, naive clustering will simply group together languages with similar properties irrespective of the specific processes that have actually *made* them similar. Instead, we seek a method that infers the relative role of contact, as opposed to the other processes, in creating similarities between languages. Here, we propose sBayes, a Bayesian mixture model that weighs the respective contributions of contact and the confounding effects from inheritance and universal preference in accounting for the similarities between languages in space. While the model was primarily developed for linguistic data and we frame our discussion in terms of language contact, sBayes is applicable to a broader range of cultural evolution data. It is available as an open-source Python 3 package on https://github.com/derpetermann/sbayes, together with installation guidelines, a manual and case studies.

### 1.1. Related work

The modern study of linguistic areas goes back to the early 20th century [22–25]. The bulk of research since then has been qualitative in nature, but recently more quantitatively oriented approaches have been developed. We discuss the history of this strand of research in §S1 of the electronic supplementary material. We conclude that a principled quantitative approach for finding contact areas is still missing, in particular one that takes into account both the process that leads to contact effects and the influence of confounding effects. A first approach to tackle this research gap was presented in [26], where a non-parametric Bayesian model was applied to reconstruct language areas. The approach recovers areal and phylogenetic effects without distinguishing universal preference and inheritance. A related idea was presented in [27], where an autologistic model together with family and neighbour graphs was used to assess the influence of inheritance and areality on cultural macroevolution in North America. The model does not itself infer areas but instead assumes the spatial influence to happen within a fixed radius of 175 km. The approach was later extended to infer latent areas from language data [28]. A somewhat different approach is proposed in [29]: based on prior knowledge, a set of languages is assigned to a potential contact area—a ‘core’. Then, a naive Bayes classifier evaluates whether other languages belong to the core or to a control set, that is, languages unlikely to have been in contact with the core. The same authors also proposed a relaxed admixture model to detect language contact [30]. This mixture model locally detects borrowings between pairs of language but does not reflect the possibility of larger contact areas.

Our method is inspired by these approaches, but, in contrast to them, it explicitly infers the assignment of languages to a contact area from the data: areas are allowed to take any possible shape and size, and they are not constrained to a pre-defined sphere of influence. Instead, a geographical prior can be used to enforce spatial coherence, and, thus, model the influence of geography. Moreover, the model controls for the two confounders of inheritance and universal preference, ensuring that only contact signals are picked up.

### 1.2. Contact areas

**Figure 1.**
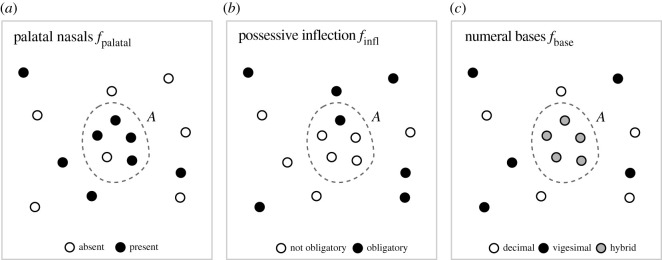
Area of shared history. In the area *A*, features (*a*) *f*_palatal_, (*b*) *f*_infl_ and (*c*) *f*_base_ (dashed-line polygon) follow a distribution with low entropy, which differs from the distribution outside of *A*. Note that the features only serve illustration here; for definitions and actual distributions, see the World Atlas of Language Structure [31].

We provide a data-driven characterization of contact areas, which builds on linguistic features, that is, structural properties of language describing one aspect of cross-linguistic diversity (as e.g. found in [31]). Consider a set of languages *L* = {*l*_1_, *l*_2_, …}, for which we study the feature *f*_palatal_, the presence and absence of palatal nasals, an item of the phonological inventory. Suppose further that there is an area *A* where palatal nasals are present in all languages, while they are commonly absent everywhere else. Universal preference fails to explain why languages in *A* have palatal nasals. We might conclude that we found evidence of some form of shared history, either due to inheritance or contact—making *A* an *area of shared history*. Clearly, this conclusion is weak: it builds on a single source of evidence and neglects chance, which becomes apparent once the distribution of a feature is less clear-cut (figure 1*a*). Inside the dashed-line polygon (*A*), languages are roughly twice as likely to have palatal nasals than outside *A*. Languages inside the polygon are similar and universal preference does not explain why. And yet, it seems arbitrary to conclude that *A* shows shared history. All the same, it seems equally arbitrary to simply disregard the similarity in *A* altogether.

A standard response is to consider additional, independent features that reinforce or weaken the similarities observed for a single feature. Suppose we also study the grammatical feature *f*_infl_, the presence and absence of obligatory possessive inflection and the lexical feature *f*_base_, the type of base system used for expressing numerals. For most languages in *A*, possessive inflection is not obligatory (figure 1*b*). Moreover, all languages in *A* use the same hybrid vigesimal–decimal base system (figure 1*c*). Each additional feature reinforces the signal observed for palatal nasals. More formally, across all three features languages in *A* have low (Shannon) entropy, i.e. they are similar and thus predictable and differ from the confounder, i.e. they cannot be explained by universal preference. This leads us to the following property: in an area of shared history *A*, independent features {*f*_1_, *f*_2_, …} follow a distribution with low entropy, which differs from the distribution expected from the confounding effect of universal preference.

This property ensures that a random accumulation of universally preferred features is not mistaken for shared history. The definition is largely impartial to the argument that preferred features are also more likely inherited and shared. For example, subject-before-object orders are universally preferred over object-before-subject orders [32,33] but the global distribution still shows geographical structure: some areas, such as Eurasia, Africa, or Papua New Guinea, show an even stronger preference than the worldwide norm. Thus, even universally preferred patterns can provide evidence for an area.

Areas of shared evolution separate unspecified shared history from universal preference, but they do not distinguish between contact and inheritance: features in *A* could have been passed on from neighbours or they could have been inherited. How can we account for the confounding effect of inheritance and, thus, isolate similarities due to contact?

**Figure 2.**
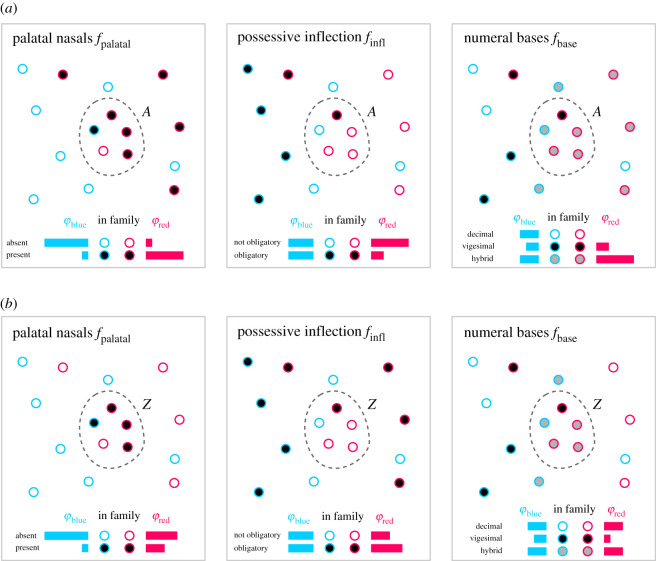
Contact areas. In the areas *A* and *Z* (dashed-line polygons) features *f*_palatal_, *f*_infl_ and *f*_base_ follow a distribution with low entropy, which differs from the distribution outside the polygons. The blue and red horizontal bars show how common a feature is in each family. (*a*) The distribution in *A* largely matches the distribution in family φ_red_. *A* can be explained by inheritance and is not a contact area. (*b*) The distribution in *Z* does not match the distribution in φ_red_. Inheritance fails to account for the similarity in *Z*, which leaves contact as the remaining explanation: *Z* is a contact area.

To approach this issue, let us assume, as an example, that most languages are related to others and belong to a language family φ∈Φ, where Φ is the set of all language families. Languages in figure 2 belong to either family φ_blue_ or φ_red_. Let us further assume that there are two areas *A* and *Z* that both contain four languages from φ_red_ and one from φ_blue_. In both areas, the entropy of each linguistic feature is lower in *A* and *Z* than is the case outside, in the entire set of languages. However, all languages in *A* have features that are also common in φ_red_ (figure 2*a*), i.e. *f*_palatal_ is present in the area and in the red family, *f*_infl_ is absent in both, and *f*_base_ is hybrid in both. This is not true for *Z*. Features in *Z* are relatively uncommon in φ_red_ (figure 2*b*), i.e. *f*_palatal_ is present in the area, *f*_infl_ is absent and *f*_base_ is hybrid, but there is no preference for either of these states in the red family. Taken together, inheritance explains the similarity in *A*, but it fails to explain the similarity in *Z*. Thus, *Z* is a contact area, whereas *A* is not. From this, we establish the following property of contact areas: in a contact area *Z*, independent features {*f*_1_, *f*_2_, …} follow a distribution with low entropy, which differs from the distribution expected from the confounding effect of universal preference. Moreover, the distribution in *Z* also differs from the distribution in families Φ and, thus, cannot be explained by the confounding effect of inheritance. Based on this property we introduce sBayes, an algorithm to find contact areas on the basis of language data.

## 2. Material and methods

sBayes requires features to be categorical. A feature *f* is assumed to have *N*_*f*_ discrete, mutually exclusive states

2.1 $$S_{f}=\{s_{1},\ldots ,s_{N_{f}}\},$$

where $S_{f}$ is the set of states and $s_{1},\ldots ,s_{N_{f}}$ are the state labels. For example, palatal nasals have two states, they can be present or absent: $S_{\mathrm{palatal}}=\{\text{present, absent}\}$. Ideally, each state is self-contained and carries explicit information about shared history, which is the case for $S_{\mathrm{base}}=\{\text{decimal, hybrid, vigesimal}\}$, but less so for $S_{\mathrm{base}}=\{\text{decimal, vigesimal, other}\}$, since the state *other* does not refer to a base system with a clear scenario of how it arises and decays.

### 2.1. Likelihood

The model aims to identify effects that predict why feature *f* in language *l* has state *s*. sBayes proposes three effects and defines a likelihood function for each:

— Likelihood for universal preference (*P*_universal_): the state is universally preferred.
— Likelihood for inheritance (*P*_inherit_): the language belongs to family *ϕ*(*l*) and the state was inherited from related ancestral languages in the family.
— Likelihood for contact (*P*_contact_): the language belongs to area *Z*(*l*) and the state was adopted through contact in the area.

sBayes models each feature as coming from a distribution that is a weighted mixture of universal preference, inheritance and contact. The unknown weights—*w*_universal_, *w*_inherit_ and *w*_contact_—quantify the contribution of each of these three effects. For a single language *l*, which is part of a family *ϕ*(*l*) and an area *Z*(*l*), we define the probability of feature *f* being in state *s* as the following mixture likelihood:

2.2 $$\begin{array}{rl} P(X_{l,f} & =s|Z,w,\alpha ,\beta ,\gamma )=w_{\mathrm{universal},f}\cdot P_{\mathrm{universal}}(X_{l,f}=s|\alpha_{f})\\ & \;+w_{\mathrm{inherit},f}\cdot P_{\mathrm{inherit}}(X_{l,f}=s|\beta_{\;f,\phi (l)})\\ & \;+w_{\mathrm{contact},f}\cdot P_{\mathrm{contact}}(X_{l,f}=s|\gamma_{\;f,Z(l)}). \end{array}$$

The mixture components—*P*_universal_, *P*_inherit_ and *P*_contact_—are categorical distributions parameterized by probability vectors *α*_*f*_, *β*_*f*,*ϕ*(*l*)_ and *γ*_*f*,*Z*(*l*)_. That is, the probability of observing state *s* in feature *f* is *α*_*f*,*s*_ if it is the result of universal preference, *β*_*f*,*ϕ*(*l*),*s*_ if it was inherited in family *ϕ*(*l*) and *γ*_*f*,*Z*(*l*),*s*_ if it was acquired through contact in area *Z*(*l*). While the assignment of languages to families is fixed, the assignment of languages to areas is inferred from the data. sBayes allows for multiple contact areas $Z=\{Z_{1},\ldots ,Z_{K}\}$, each with their own set of areal probability vectors. A detailed explanation of all mixture components together with examples can be found in §S2 of the electronic supplementary material.

The weights *w*_*f*_ = [*w*_universal,*f*_, *w*_inherit,*f*_, *w*_contact,*f*_] model the influence of each component on a feature:

2.3 $$w_{\mathrm{universal},f},\;w_{\mathrm{inherit},f},\;w_{\mathrm{contact},f}\geq 0$$

and

2.4 $$w_{\mathrm{universal},f}+w_{\mathrm{inherit},f}+w_{\mathrm{contact},f}=1.$$

For languages not assigned to a contact area, the contact weight is set to zero and the other weights are re-normalized accordingly. We describe this normalization and the resulting likelihood in §S2 of the electronic supplementary material.

The mixture model combines the likelihood for universal preference, inheritance and contact and their weights across all languages. The model has parameters Θ={Z,α,β,γ,w}, which are evaluated against the data *D*, that is, the states of all features in all languages. The likelihood of the whole model for the given data is the joint probability of the observed feature values *D*_*l*,*f*_ over languages *l* ∈ *L* and features *f* ∈ *F*, given Θ:

2.5 $$P(D|\Theta )=\prod_{l\in L}\prod_{\;f\in F}P(X_{l,f}=D_{l,f}|\Theta ).$$

### 2.2. Model intuition

sBayes preferentially samples areas with high likelihood values. This is the case if estimates for the areal probability vector, *γ*_*f*,*Z*(*l*)_,

— fit the data,
— have low entropy, and
— differ from the probability vectors of the confounders.

**Figure 3.**
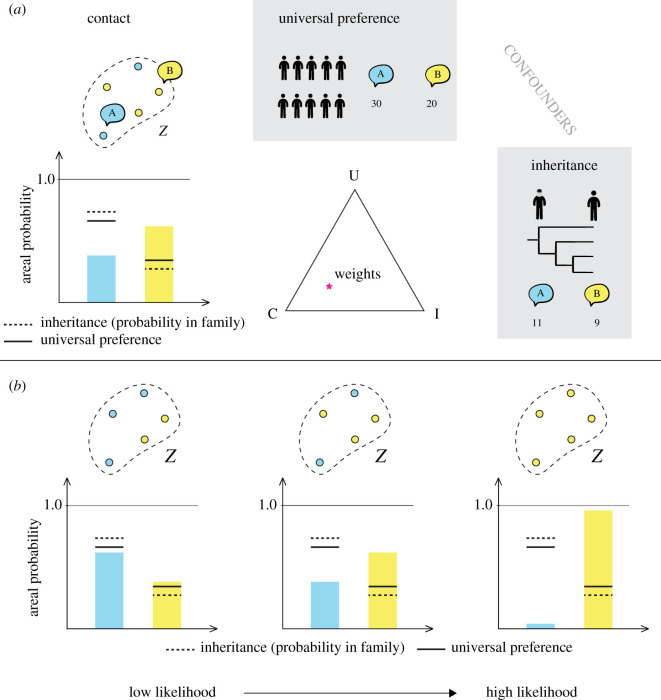
(*a*) The languages in area *Z* are explained better by contact than universal preference and inheritance. The weights vector (pink star) leans towards contact. (*b*) The likelihood of the model is highest when the areal probability vector has low entropy (i.e. features in *Z* are similar) and when it differs from the confounders.

Figure 3*a* illustrates how sBayes evaluates evidence for contact for a single feature with two states *A* (blue) and *B* (yellow). The distribution of the feature in the proposed area *Z* has low entropy (blue and yellow columns) and differs from the distribution of the two confounders—universal preference (solid black line) and inheritance (dashed black line). This pulls the weights vector (pink star) towards contact. Figure 3*b* shows that given the same confounding effect the likelihood increases with increasing entropy in area *Z*. sBayes avoids areas where universal preference and inheritance explain the similarity in the data equally well or even better than contact, but instead picks up areas for which the confounders do not provide an adequate explanation, given that their entropy is low.

### 2.3. Prior

In sBayes, priors must be defined for the mixture weights and the probability vectors of the categorical distributions on the one hand, and the assignment of languages to areas on the other. sBayes uses Dirichlet priors for the weights and the probability vectors and purpose-built geo-priors for the assignment of languages to areas:

2.6 $$P(\Theta )=\underset{\mathrm{geo-prior}}{\underbrace{P(Z)}}\cdot \;\underset{\mathrm{Dirichlet}\;\mathrm{prior}}{\underbrace{P(\alpha )\cdot P(\beta )\cdot P(\gamma )\cdot P(w)}}.$$

Both the weights *w*_*f*_ and the vectors *α*_*f*_, *β*_*f*_, *γ*_*f*_ parameterize a categorical distribution: they are bounded between [0, 1] and sum to 1, which motivates the use of a Dirichlet prior:

2.7 $$\alpha_{f}∼\mathrm{Dir}(\psi_{f}^{\left(\alpha \right)})\;\beta_{\;f,\phi }∼\mathrm{Dir}(\psi_{\;f,\phi }^{\left(\beta \right)})\;\gamma_{f}∼\mathrm{Dir}(\psi_{\;f}^{\left(\gamma \right)})$$

and

2.8 $$w_{f}∼\mathrm{Dir}(\psi_{\;f}^{\left(w\right)}).$$

The default prior is uniform, i.e. we set $\psi_{\cdot }^{\left(\cdot \right)}=(1,\ldots ,1)$ for all weights and probability vectors.

In other words, any of the *N*_*f*_ states and any of the three weights are equally likely *a priori*. While this invariance seems reasonable for the weights, it might not always be appropriate for the probability vectors: *α*_*f*_ allows the model to learn which states are universally preferred, and *β*_*f*,*ϕ*_ which states are inherited in family *ϕ*. The more a state is preferred universally or in a family, the less likely a similar occurrence in *Z* is regarded as evidence for contact. However, what is rare in our sample (i.e. our study area) might be abundant outside and vice versa.

In an ideal setting, sBayes would be applied to a global sample of languages, making it possible to infer universal preferences directly from the data (in which case we would recommend using the uniform prior). When this is not possible, preference may be incorporated in the form of an empirical prior. The prior allows us to express specific knowledge about universal preferences before seeing the data. In the Dirichlet distribution, the parameters *ψ*_*f*,*n*_ can be thought of as pseudocounts for each of the *N*_*f*_ states, reflecting prior knowledge or assumptions:

2.9 $$\psi_{\;f,n}^{\left(\alpha \right)}=1+\mu_{n}\cdot \rho \;\mathrm{for}\;n\in 1,\ldots ,\;N_{f}.$$

In equation (2.9), *μ*_*n*_ is the prior probability of state *s*_*n*_ and defines the mean of the prior distribution, while *ρ* gives the precision or inverse variance. A large *ρ* implies a strong prior with low variance. An informative prior for inheritance in family *ϕ* is defined analogously. In §S3.1 of the electronic supplementary material, we illustrate how a biased sample might lead to biased estimates for universal preference and we provide an example for an empirically informed prior.

Each language *l* is geographically situated: it has a spatial location, that is, a unique point in geographical space (if we assume languages to be represented by their centre of gravity). The geo-prior models the *a priori* probability of languages in an area to be in contact, given their spatial locations. sBayes employs two types of geo-priors:

— a *uniform* geo-prior and
— a *cost-based* geo-prior.

The *uniform* geo-prior assumes all areas to be equally likely, irrespective of their spatial locations, whereas the *cost-based* geo-prior builds on the assumption that close languages are more likely to be in contact than distant ones. Distance is modelled as a cost function *C*, which assigns a non-negative value *c*_*i*,*j*_ to each pair of locations *i* and *j*. Costs can be expressed by the Euclidean distance, great-circle distance, hiking effort, travel times, or any other meaningful property quantifying the effort to traverse geographical space. Since costs are used to delineate contact areas, they are assumed to be symmetric, hence *c*_*i*,*j*_ = *c*_*j*,*i*_. For cost functions where this is not immediately satisfied the cost values can be made symmetric, e.g. by averaging the original costs.

sBayes applies a linkage criterion to connect all languages in area *Z*_*k*_. The default criterion is the minimum spanning tree *T*_*k*_, which connects all languages in *Z*_*k*_ with the minimum possible costs (red and blue lines in figure S1*b*, electronic supplementary material). Other linkage criteria are discussed in §4. *T*_*k*_ quantifies the *least* effort necessary for speakers in *Z*_*k*_ to *physically meet* given the particular cost function used. We define the cost of an area as the average cost over all edges in the minimum spanning tree

2.10 $$c_{k}\;:=\sum_{i,j\in T_{k}}\frac{c_{i,j}}{\left|T_{k}\right|},$$

and let the prior probability decrease exponentially as the average cost increases:

2.11 $$P_{\mathrm{geo}}(Z_{k}|C)\propto e^{-\lambda \;c_{k}}.$$

The parameter *λ* defines the rate at which the probability decreases. A large *λ* results in a strong geo-prior: distant languages with high costs have very low prior probability to allow for contact. When *λ* is small, the exponential function becomes flat and the geo-prior approaches a uniform distribution (figure S1*b*, electronic supplementary material). Using the average (rather than the sum) to define the cost of an area ensures that this prior is agnostic to the number of languages in *Z*_*k*_. The geo-prior not only expresses our belief that spatial proximity leads to contact but also expresses our confidence in the present-day locations of languages, which might have been different just a few hundred years ago.

Note that equation (2.11) only defines a prior on a single area, while sBayes models multiple areas and assumes that they are disjoint. For multiple areas, we define the joint prior by truncating the product of the independent priors to the set of all non-overlapping areas:

2.12 $$\begin{array}{r} P_{\mathrm{geo}}(Z|C)\propto \left\{ \begin{array}{cc} \prod_{k=1}^{K}\;e^{-\lambda \;c_{k}}, & \text{if all areas in}\;Z\;\text{are disjoint}\\ 0, & \mathrm{otherwise}. \end{array}\right. \end{array}$$

In addition to the geo-prior, there are two implicit parameters relating to the prior probability of contact areas: the size of an area in terms of number of languages, *m*_*k*_ :=|*Z*_*k*_| for *k* ∈ {1, …, *K*}, and the number of areas, *K*. The prior for *m*_*k*_ is discussed in §S3 of the electronic supplementary material. There is no prior for *K*. Instead, we run the model iteratively, increase the number of areas per run and compare the performance across *K* in postprocessing (see §2.5).

### 2.4. Posterior

The posterior of the model is proportional to the likelihood times the prior:

2.13 P(Θ|D)∝P(D|Θ)⋅P(Θ).

Section S5 of the electronic supplementary material explains how sBayes samples from the posterior distribution P(Θ|D) to identify potential contact areas. sBayes employs a Markov chain Monte Carlo (MCMC) sampler with two types of proposal distributions: a Dirichlet proposal distribution for weights and probability vectors and a discrete, spatially informed proposal distribution for areas.

### 2.5. Number of areas

With more areas sBayes will find it easier to explain the variance in the data. However, each area requires additional parameters, resulting in a more complex model and higher uncertainty in the posterior. sBayes employs the *deviance information criterion* (DIC) to find a balance between fit and complexity. The DIC estimates the effective number of parameters from the uncertainty in the posterior and uses it to penalize the goodness of fit [34]. We run sBayes iteratively increasing the number of areas *K* and evaluate the DIC for each run. The most suitable *K* is where the DIC levels off, such that adding more areas does not improve the penalized goodness of fit. The DIC has been found to outperform competing approaches for identifying the optimal number of clusters in a comparable Bayesian clustering procedure [35]. We show that the DIC correctly reports the true number of areas in simulated data (§S7 of the electronic supplementary material). However, the DIC is not part of the core methodology and can be replaced with other model selection criteria, e.g. the WAIC [36] or PSIS-LOO [37].

Once a suitable *K* has been identified, areas are ranked according to their relative posterior probability in post-processing (see §S4, electronic supplementary material).

## 3. Results

For all experiments, we ran sBayes with 3 million steps, of which the first 20% were discarded as burn-in. We retained 10 000 samples from the posterior and used Tracer [38] to assess the effective sample size and convergence.

### 3.1. Simulation study

Before applying sBayes to real-world data, we performed a simulation study to verify that the algorithm correctly samples from the posterior distribution under model assumptions. We assigned 951 languages to random locations in space and simulated 30 features for each to model universal preference. All features were generated according to a categorical distribution with two, three, or four states. In §S7 of the electronic supplementary material, we show that the simulated distributions seem plausible when compared to the empirical distributions of the case studies. We carried out four experiments:

— *Experiment 1* correctly identified contact areas differing in shape, size and strength of the signal.
— *Experiment 2* distinguished between similarity due to inheritance and due to horizontal transfer, separating contact effects from inheritance in a family.
— *Experiment 3* correctly estimated the number of contact areas.
— *Experiment 4* used empirically informed priors to robustly infer contact areas even for small and biased samples.

Experiment 2 will be explained in more detail below. All remaining simulation experiments can be found in §S7 of the electronic supplementary material. Experiment 2 demonstrates that sBayes distinguishes between similarities due to inheritance and those due to contact. We assigned some of the simulated languages to a common language family and some to a contact area. We simulated shared ancestry in the family and contact in the area with different categorical distributions. The entropy for inheritance was set to be lower than that of contact, i.e. the signal for shared ancestry was assumed to be stronger. Finally, we simulated weights controlling the influence of each effect. Then, sBayes was run with two different setups. In the first setup, the information about common ancestry was not passed to the algorithm. sBayes incorrectly attributes the similarity in the family to contact. Assuming a single contact area (*K* = 1), the posterior of *Z*_1_ overlaps with the simulated language family, but misses out on the weaker simulated contact area (figure S5*a*, electronic supplementary material). In the second setup, inheritance was modelled at the family level and passed to the algorithm. Now, sBayes was able to learn that the similarity in the family was due to inheritance. The posterior correctly returns the simulated contact area (figure S5*b*, electronic supplementary material).

**Figure 4.**
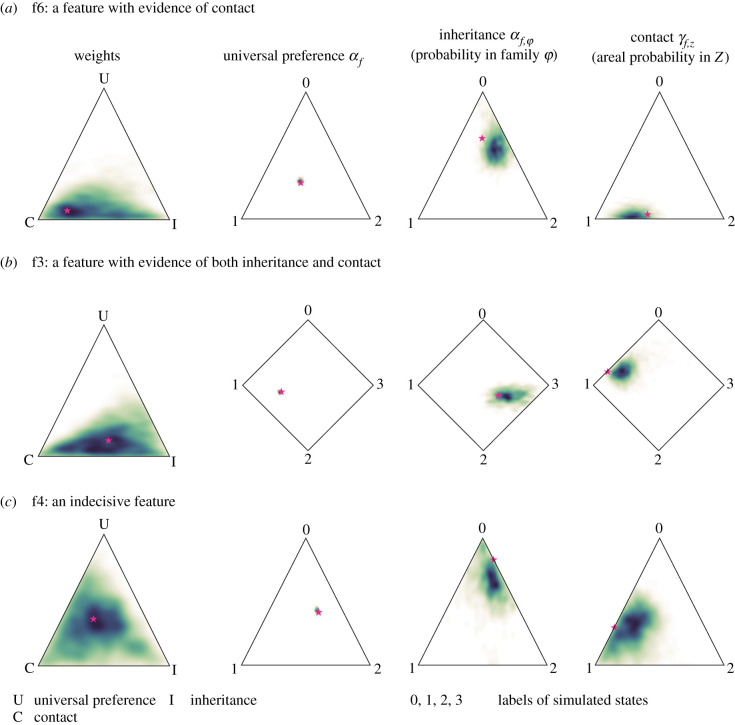
Simulated and reconstructed weights and states (U: universal preference; I: probability of inheritance in family *ϕ*; C: probability of a contact effect in area *Z*) for three features (f6, f3 and f4). The heat map shows the probability density of the posterior distribution. The pink star marks the ground truth value, i.e. the simulated weights or states. Feature f6 provides evidence of contact (*a*), f3 of inheritance and contact (*b*) and f4 is indecisive (*c*).

sBayes not only finds contact areas, but also infers the influence of each feature to delineate them. Figure 4 shows the simulated values for universal preference, inheritance in the family, and contact in *Z*_1_ (pink star) for three features, and their inferred posterior distribution (heat map ranging from yellow to dark blue). Feature f6 (figure 4*a*) is strongly shared in *Z*_1_; both the simulated and inferred weights lean towards contact. In the area, most languages have either state 1 or 2. In the family, state 0 is preferred. Universally, there is no preference for either state. Feature f3 (figure 4*b*) is both inherited in the family and shared in the area. The simulated and inferred weights lie between contact and inheritance. Feature f4 (figure 4*c*) is indecisive. The simulated weights lie in the centre, and the inferred estimates scatter across the entire probability simplex.

### 3.2. Case study: western South America

Western South America is characterized by extreme genealogical diversity. At the same time, the languages in this region share a number of structural linguistic features that have been argued to result from contact. A major split between two cultural macro-areas of linguistic diffusion, the Andes and the Amazon, has been proposed [39–42]. This has led to lists of ‘Andean’ and ‘Amazonian’ linguistic contact features. More recent work, although generally recognizing weak areality for the macro-areas, has focused on more circumscribed contact areas within the Andean and Amazonian macro-areas as resulting more clearly from contact [29,43,44]. On the basis of this, we expect to find the strongest signals to be pointing towards these smaller subareas, with a secondary effect in that these smaller areas are still by-and-large confined to either of the two macro-areas.

**Figure 5.**
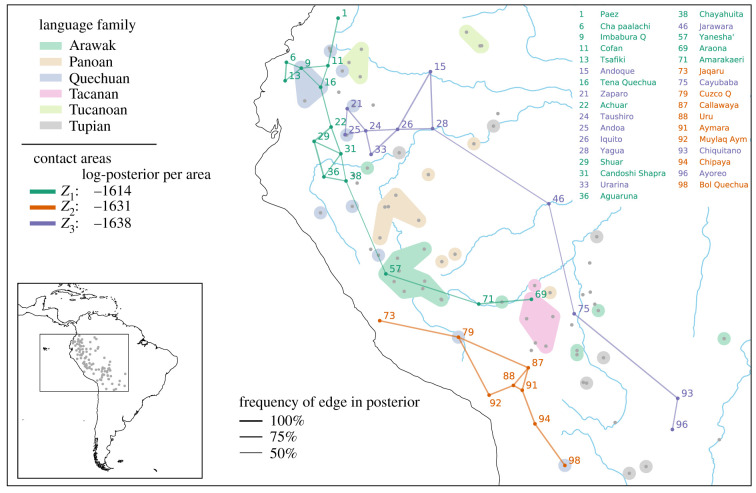
Contact areas in western South America. The posterior distribution consists of contact areas *Z*_1_, *Z*_2_ and *Z*_3_ (connected by green, orange and purple lines), ordered by posterior probability. The grey dots indicate the spatial locations of all languages in the sample, the shaded areas represent the six main language families. Languages in each area are connected with a Gabriel graph [45]; line thickness corresponds to the frequency of an edge in the posterior (how often are two adjacent languages together in the same area?).

The dataset used for the case study consists of 100 languages presently spoken in the western Amazon basin and adjacent Andean highlands (figure S9, electronic supplementary material). The 100 languages were coded for 36 features of grammar, many of which are thought of as either ‘Andean’ or ‘Amazonian’ (table S2, electronic supplementary material). The prior for universal preference was derived from a stratified global sample (86 languages from different language families spread uniformly over the globe). The mean of the Dirichlet prior was set equal to the mean of the stratified sample. The precision was set to 10, yielding a weakly informative prior. Inheritance was modelled for families with at least five members: Arawak, Panoan, Quechuan, Tacanan, Tucanoan and Tupian. A prior for each family was derived from 37 languages outside the sample analogously to the universal prior, except for Tacanan, for which all (known) members were in the sample and a uniform prior was used instead. The geo-prior was set to be uniform. Figure 5 shows the results of the experiment. Language families are shown by shaded areas, contact areas by coloured lines. We ran the analysis iteratively, increasing the number of areas per run. The DIC starts to level off for *K* = 3, suggesting three salient contact areas in the data (figure S10, electronic supplementary material).

The northern part of *Z*_1_ has likely been an area of interethnic interaction for a long time, connected to the sphere of influence of the Chibcha family [46–48], and smaller-scale interactions into the lowlands (e.g. [49–51]). Of the geographically more remote languages, Yanesha’ and Araona (or more generally the Tacanan family) have known historical contact relations with Quechuan languages [52]. Moreover, it has been observed that Yanesha’ shares features with the northern cluster [51]. Amarakaeri is a relatively recent arrival in the foothills, with looser ties to the Incas [46,52]. The contact features contributing most to the northern area are generally associated with Andean languages [41,42] (figure S12, electronic supplementary material).

Area *Z*_2_ corresponds to a well-known case of intensive language contact between Aymaran and Quechuan languages and, more peripherally, the Uru-Chipaya family [42,52]. The most likely contact features in our analysis correspond to known Andean features, mostly phonological (figure S13, electronic supplementary material).

Areas *Z*_1_ and *Z*_2_ roughly correspond to the northern and southern central Andes, respectively (with some incursions into the lowlands). This is consistent with recent results [29,44], which suggest that the Andes consist of ‘two distinguishable but interlocking linguistic areas, one northern and one southern’ [44].

The Amazonian-based area *Z*_3_ is spread over a large territory, which may be due to the fact that Amazonian languages, generally speaking, lack a number of features that are characteristic for Andean languages. This is corroborated by the most contributing features which mark the absence of typical Andean characteristics (figure S14, electronic supplementary material). The densest part of this area, however, may be connected to the idea of a larger trade area around the Marañon River [44,53], ultimately connected to the north-western part of a vast trade area [54,55]. A contributing reason for the connection between the northern and southern clusters of area *Z*_3_ may be the fact that the two largest families of the continent, Arawak and Tupian, have branches that extend into the northwest Amazon as well as the Madeira-Guaporé-Mamoré area in the south.

Concluding, we do indeed find some of the proposed smaller Andean and Amazonian contact areas, as well as possibly some long-distance signals in the Amazon area. We also find some evidence of highland–lowland contact, which is in line with areal–typological work that encompasses both macro-areas [56–59]. All of this is largely consistent with the literature, although not all proposed contacts receive equally clear support, such as the Guaporé-Mamoré area [60]. This may be due to the fact that the contact signal of other areas is stronger.

### 3.3. Case study: Balkans

The Balkan peninsula is one of the linguistic areas that was proposed earliest [61] and received intensive discussion (for a historical overview and critical assessment of the key concepts see [62,63]). It contrasts with the South American case in its much smaller size and the reduced diversity of language families. The obvious impact of inheritance for many of the similarities between the varieties tends to be dismissed, often referring to the long time since speciation [64]. More recently, it has been proposed that instead of one single area, the Balkan peninsula actually features smaller clusters of convergence [63]. On the basis of this, we expect sBayes to report a single large Balkan area when the number of areas is set to one. At the same time, we expect that several salient subareas emerge when the number of areas is increased, subdividing the Balkans into smaller clusters. Specifically, we expect to find a subarea near lake Ohrid and lake Prespa, at the border between Albania, North Macedonia and Greece [65,66].

**Figure 6.**
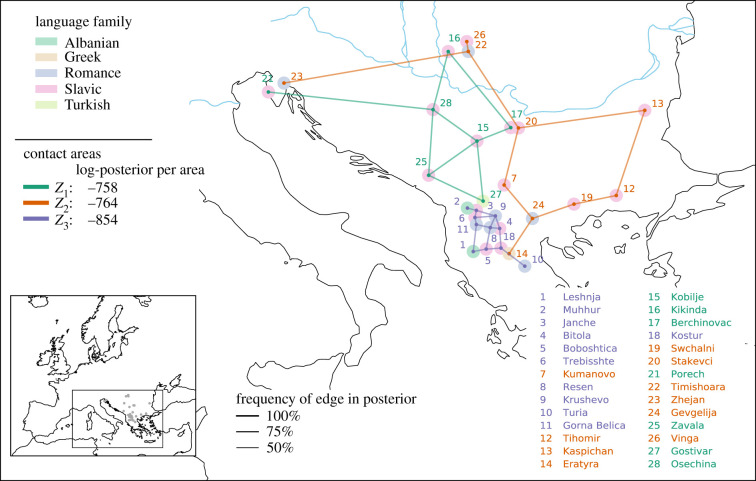
Contact areas in the Balkans. The posterior distribution consists of contact areas *Z*_1_, *Z*_2_ and *Z*_3_ (connected by green, orange and purple lines) ordered by posterior probability. The shaded circles represent the sub-clades and language families. Languages in an area are connected with a Gabriel graph; line thickness corresponds to the frequency of an edge in the posterior (how often are two adjacent languages together in the same area?).

The dataset consists of 30 languages and dialects situated within and outside the geographical boundaries of the Balkan peninsula: Albanian, Macedonian, Bulgarian, Torlak, Aegean Slavic, Bosnian-Croatian-Montenegrin-Serbian, Aromanian, Istroromanian, Romanian of Romania and Moldova and Balkan Turkish (figure S15, electronic supplementary material). With the exception of Turkish, they all belong to the Indo-European family. The 30 varieties were coded for 47 features from various linguistic domains (see table S3, electronic supplementary material). Inheritance was modelled at the sub-clade level for Albanian, Greek, Romance and Slavic dialects and at the family level for Turkic. We used a stratified sample of 19 European languages to model a prior for universal preference (or, in this case, Standard Average European preference). The mean of the Dirichlet prior was set equal to the mean of the European sample. The precision was set to 10, resulting in a weakly informative prior. Analogously, we collected 23 languages outside the sample to derive empirically informed priors for all sub-clades, except for Albanian, for which all members were in the sample and a uniform prior was used instead. The geo-prior was set to be uniform. Figure 6 shows the results of the experiment. Language families are shown as shaded areas, contact areas by coloured lines. We ran the analysis iteratively, increasing the number of areas per run. The DIC levels off for *K* = 3, after which it increases sharply, suggesting three areas in the data (figure S17, electronic supplementary material).

As expected, the dialects in the sample share a common history that differs from both Standard European preference and the family probability in each of the sub-clades. For *K* = 1, all dialects are assigned to one single area—except for the two Albanian dialects of Leshnja and Muhhur, and the Turkish dialect of Gostivar, which are still reasonably well explained by inheritance in the Albanian sub-clade and in the Turkic family, respectively (figure S16, electronic supplementary material). This single Balkan area divides into three salient areas (figure 6), now also including the above three dialects.

Area *Z*_1_ joins different varieties of the southwestern part of the Serbo-Croatian dialect continuum. The area is distinct within the Slavic branch and—as indicated in figure S19, electronic supplementary material—is defined through a lack of features that would traditionally be expected in the Balkan Sprachbund [67,68]. Dialects in *Z*_1_ had almost no contact with Albanian and Romance/Aromanian and were not exposed to the processes of language convergence observed in areas *Z*_2_ and *Z*_3_. The fact that Gostivar Turkish belongs to *Z*_1_ indicates that it has converged with these varieties in certain respects.

Area *Z*_2_ includes the Greek variety of Eratyra, the Meglenoromanian variety of Gevgelia, all Bulgarian dialects, the Romance varieties to the north of the Danube, i.e. Slavic dialects spoken in the Aegean, Slavic dialects in a Romance surrounding and Romance dialects in a Slavic surrounding. The area shows Romance–Slavic and Slavic–Greek contacts. Interestingly, some of the defining features (F30, F33, F38; figure S20, electronic supplementary material) are characteristic of Albanian dialects and are also shared in *Z*_3_, suggesting contact between the two areas. This is also reflected when running sBayes with *K* = 2, in which case *Z*_2_ and *Z*_3_ are merged into a single large area.

Area *Z*_3_ comprises all Albanian and Aromanian, as well as the western Macedonian Slavic dialects. The area shows intense contact and multilingualism, characterized by a set of properties for which a contact explanation is the most probable one (figure S21, electronic supplementary material). This corresponds to what is known from traditional studies of the Balkan area, which identify the area around lake Ohrid and along the border between today’s Albania and North Macedonia as the centre of areal innovations [65,66].

Overall, Slavic varieties partake in all three areas. sBayes clearly divides West South Slavic and East South Slavic. The former constitutes an area mainly by its divergence within the Slavic branch as a result of dialect contacts. Whether these varieties are also part of another convergence zone, e.g. with the languages of the Austro-Hungarian Empire, remains to be investigated with additional data. East South Slavic is affected by different contact situations: with Romance and Greek in *Z*_2_, with Romance and Albanian in *Z*_3_. In this way, a historical interpretation of the three areas seems possible: *Z*_1_ is the oldest area of internal South Slavic dialect contact (Turkish joining later), *Z*_2_ shows contact fostered by the Byzantine Empire, while *Z*_3_ reflects contact triggered within the Ottoman Empire. In any case, contact with Albanian emerges as the crucial element responsible for the specific Balkan convergence processes in *Z*_3_. In sum, the three areas largely confirm what is known from traditional studies, albeit on a strictly empirical basis and disclosing the relevant premises.

## 4. Discussion

We presented sBayes, a Bayesian clustering algorithm to identify areas with similar entities while accounting for confounders. Specifically, we tailored the approach to language data and identified areas of language contact, while accounting for universal preference and inheritance. We tested the approach on simulated data and performed two case studies on real-world language data in South America and in the Balkans. The results suggests that sBayes successfully detects these areas, and it can therefore be used for testing other hypothesized contact areas or for searching them in a bottom-up manner, at any scale. In what follows we discuss the assumptions, extensions and limitations of any such application.

### 4.1. Model assumptions and diagnostics

Our model assumes that contact leaves behind traces in extant languages in the form of areas, which emerge once the more salient traces of confounding effects have been properly accounted for. Specifically, the mixture model assumes that each feature in each language is explained probabilistically by three effects: universal preference, inheritance in a family and contact in an area. sBayes iteratively proposes areas and evaluates them against the data. Areas have a high likelihood for contact if they comprise similar features which cannot be equally well explained by universal preference and inheritance. There are no assumptions about any of the properties of contact areas, such as their shape, size or number, whether they comprise close or distant languages, or cover contiguous or disconnected regions in space. The algorithm learns these properties from the data, potentially guided by informative (geographical) priors. Likewise, sBayes is agnostic to features and their relationship to borrowing. *A priori*, all features are treated as equal and independent evidence. Proposing and evaluating contact areas in turn, the algorithm learns which features are better explained by each of the three effects. In this sense, the analysis is data-driven: only sufficient, informative and independent features provide a robust statistical signal to delineate contact areas.

sBayes is one of several recent statistical models for analysing contact in language data. Our focus lies on the spatial signal: the model infers contact areas from language data without superimposing a spatial neighbourhood effect *a priori*. The model recovers past contact across language families even in cases when the current geographical locations of these contact languages are far apart. This spatial flexibility is achieved by reducing the complexity of the clustering: in order to keep the model simple and clustering tractable, we require that one language belongs to one area at a time and that areas cannot overlap. Complementary statistical models have shown that these assumptions can be relaxed, for example when the spatial influence is defined on extra-linguistic grounds [69], when clustering is applied to languages in a single family [28,29,70,71] or when it is applied to recover unspecified shared ancestry [72]. In future work, it will be interesting to explore whether statistical inference with sBayes is still tractable when the model supports probabilistic assignments of languages to areas and allows areas to overlap, while still inferring areality from the data rather than predicting it from extra-linguistic evidence.

sBayes does not replace expert knowledge in defining the features, the confounders (e.g. the families), the priors, and the spatial locations and in interpreting the results in an anthropological and historical context. In the absence of salient contact areas in the data, sBayes might group together outlier languages that are poorly explained by either of the confounders. sBayes provides statistics and measures to detect such potentially spurious areas in the posterior. MCMC diagnostics assess whether sampling has converged to a stable, stationary distribution and whether the posterior contains sufficient independent samples (§S5, electronic supplementary material). Measures of model fit evaluate the evidence for contact in the posterior. Spurious areas have a high entropy and a low likelihood, resulting in a high DIC. Priors account for biased data and enforce spatial plausibility. However, statistics and priors can only address the internal validity of the model. Potentially spurious areas can still arise because of misspecified confounders, e.g. the algorithm returns a language family that was not included in the model, or because of redundant features that encode very similar or identical linguistic concepts. Therefore, the most important sanity check comes from the domain experts who pick the features, model the confounders and interpret the results.

### 4.2. Modelling confounders

In order for the algorithm to function properly, all confounding effects must be modelled correctly and completely. Specifically, sBayes assumes that—once universal preference and inheritance have been accounted for—the remaining similarity in the data is due to contact. We will briefly discuss the confounders currently considered in the model and give an outlook on future extensions.

Universal preference helps the algorithm to establish a baseline for chance. sBayes learns how often a feature is expected in extant languages. There are different conceptual approaches for estimating universal preference, yielding a nuanced interpretation for contact and contact areas. When the baseline is derived from the data alone, it encodes preference in the study area. This is appropriate for a sufficiently large and balanced sample, while small and unbalanced samples are likely to yield a biased baseline, resulting in biased areas. For example, the 30 languages coded for in the Balkans case study are similar precisely because they share a common history, in which case it makes sense to inform the baseline with an empirical prior encoding preferences outside the biased sample.

**Figure 7.**
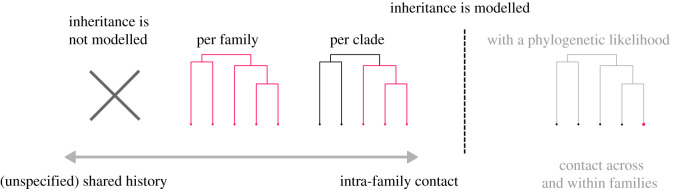
Information about inheritance can be modelled in sBayes at different levels (highlighted in red), causing the algorithm to pick up different contact signals, which range from (unspecified) shared history to intra-family contact. For future versions, a phylogenetic likelihood could model inheritance as a hierarchical process and reveal nuanced traces of contact. In this phylogenetic model, the probability of each state can be estimated separately for each of the tips in the tree, i.e. for each of the extant languages (red dot).

Inheritance helps the algorithm to establish a baseline for chance in a family. There are different conceptual levels (and levels of granularity) at which information about common ancestry can be passed to sBayes (figure 7). When no information about common ancestry is available, the model does not distinguish between inheritance and contact. Instead, it identifies areas of unspecified shared history, i.e. subsets of languages with similar features whose similarity is only poorly explained by universal preference and derives from a web of inheritance and contact, or both together. When common ancestry is modelled at the family level, sBayes estimates one set of probability vectors per language family, picking up contact across families, but not within. When modelled at the clade level, sBayes estimates one set of probability vectors per sub-clade of a language family, revealing contact both across families and across clades. It is up to the analyst to define the granularity at which the phylogeny is split into clades: the finer the splits, the more the model is able to pick up contact between closely related languages. However, increasing granularity brings about decreasing statistical robustness. Too few languages per clade (<5) make it difficult to estimate robust probability vectors.

In reality, inheritance is a hierarchical process. While all languages in a family are expected to inherit some shared features, close relatives do so more than distant ones. A phylogenetic likelihood could capture this hierarchical process in a principled way.

We plan to extend sBayes and implement a tree-based likelihood whenever the user provides a phylogeny for a language family. In this model, the phylogeny would help sBayes to estimate the probability of ancestral states, for example using Felsenstein’s pruning algorithm [73]. This would result in better estimates for confounding for each language in the family, making it possible to pick up nuanced signals of contact across and within families. Ideally, information should be exchanged in both directions. While sBayes accounts for inheritance when finding contact areas, we need phylogenetic models that account for the complex interplay between inheritance and contact when reconstructing evolutionary trees. Thus, sBayes can only be a first step towards probabilistic models that can empirically infer the full complexity of linguistic evolution.

Besides universal preference and inheritance, there are other confounders that could shape the distribution of linguistic features. For example, climate [74], altitude [75], genetics [76], subsistence [77] and population size [78] have all been hypothesized to influence the human sound inventory. All of these factors could lead to parallel convergence: potentially far-away languages are exposed to the same evolutionary dynamics and, thus, evolve similarly. In its current setup, sBayes does not consider additional confounders and might interpret parallel convergence as contact. While this is unlikely to happen—parallel convergence would need to occur in several of the features in order to result in a detectable signal and we can avoid areas between unrealistically far languages with a strong geo-prior—one could also adapt sBayes to consider additional confounders. For example, to account for an additional climate confounder, we would add an effect to the mixture model, assign languages to climate regions and estimate a distribution for each. However, adding confounders requires careful consideration. Climate and contact are likely correlated: geographically close languages tend to have a similar climate and they are more likely to be in contact. Thus, a climate confounder would explain parts of the actual contact signal, which might be undesirable.

### 4.3. Testing hypothesis of spatial evolution

The geo-prior models the prior belief of areas as a function of costs to traverse geographical space: what is the probability that languages have been in contact given the distance between them? There are two different applications of the geo-prior. First, it helps to guide inference. An informed geo-prior will encourage the algorithm to delineate spatially compact areas, coinciding with traditional ideas of what constitutes a linguistic contact area. A reasonably informed geo-prior penalizes but does not exclude: if the contact signal is strong enough in remote languages, the algorithm will still report the similarities between them as areas. Second, the geo-prior can be used to test hypotheses of spatial evolution. For instance, in the dense vegetation of the Amazon rainforest contact might be more likely between languages connected by navigable waterways. One could define a model with a uniform geo-prior and one with a strong geo-prior with costs defined as canoeing distance along the river network. The marginal likelihood, e.g. approximated with a stepping stone sampler [79], could quantify the evidence of each model. Bayesian model selection [80] could determine which model is more likely given the data. In a similar way it is possible to model other prior beliefs about geography, socioeconomics, or environment and test their influence on the clustering: are emerging contact areas best explained by hiking effort, trade routes, or vegetation?

Users can also change the linkage criterion for evaluating the geo-prior. The default criterion is the minimum spanning tree, which does not necessarily assume direct contact between all languages. Instead, properties can spread from one intermediary language to the next, connecting a chain of potentially far away languages. This linkage criterion is plausible when assuming that properties spread sequentially in a network of continuous interaction, for example along trade routes. Other linkage criteria are the Delaunay triangulation [81], which connects each language to several of its neighbours, and the complete graph, which connects each language to all other languages in the area. Both criteria require more direct interaction and reflect a more compact notion of areas. In the case of the complete graph, all mutual pairs of languages in an area are required to be spatially close.

### 4.4. Applications beyond linguistics

Besides language contact, there are other domains where sBayes can be applied. Contact between groups has many more dimensions than language, which can be analysed using sBayes as long as they can be captured in the form of features. One dimension is culture: wherever people are in contact, they tend to exchange artefacts, but also cultural practices, ideas, rituals, mythology, etc. All of these types of exchange may leave traces in the anthropological and archaeological record. Although feature-based interpretations of cultural practices have been criticized [82], there is an ongoing tradition to do so (e.g. [83–85]). Studies conducted show that meaningful reconstructive models can be built on the basis of cultural features [27,83,86–88]. Moreover, the geo-prior could be used to test hypotheses of evolution in space and compare human evolution across different dimensions. Does cultural contact follow similar pathways as genetic variation? This hypothesis could be evaluated against empirical data by using spatial clusters emerging in genetic data as a geo-prior when applying sBayes to cultural data.

Potentially, the use of sBayes might also be explored to tackle other problems outside the broader domain of cultural evolution. In ecology, for example, sBayes might reveal ecological habitats while controlling for preferences due to confounders such as climate or soil patterns. In environmental science, sBayes might show toxic hotspots while controlling for known effects due to population density or traffic. In social network data, the proposed algorithm might reveal similarities across users, while controlling for socio-cultural preferences.

## Data accessibility

The data for the two case studies are available at https://github.com/derpetermann/sbayes, together with the software, the installation guidelines and a manual.

## References

[1] Schuchardt H. 1884 Dem Herrn Franz von Miklosich zum 20. Nov. 1883: Slawo-Deutsches und Slawo-Italienisches. Graz, Austria: Leuschner & Lubensky.

[2] Balthasar B. 2020 Large and ancient linguistic areas. In Language dispersal, diversification, and contact: a global perspective (eds M Crevels, P Muysken), pp. 78-101. Oxford, UK: Oxford University Press.

[3] Campbell L. 2006 Areal linguistics: a closer scrutiny. In *Linguistic areas: convergence in historical and typological perspective* (eds Y Matras, A McMahon, N Vincent), pp. 1–31. Basingstoke, UK: Palgrave MacMillan.

[4] Dahl Ö. 2001 Principles of areal typology. In *Language typology and language universals*, vol. 2 (eds M Haspelmath, E König, W Oesterreicher, W Raible). Berlin, Germany: Mouton de Gruyter.

[5] Gray R, Bryant DB, Greenhill S. 2010 On the shape and fabric of human history. Phil. Trans. R. Soc. B **365**, 3923-3933. (10.1098/rstb.2010.0162) 21041216 PMC2981918

[6] Matras Y. 2011 Explaining convergence and the formation of linguistic areas. In *Geographical typology and linguistic areas* (eds O Hieda, C König, H Nakagawa). Amsterdam, The Netherlands: John Benjamins.

[7] Muysken P. 2013 Language contact outcomes as the result of bilingual optimization strategies. Bilingualism: Language and Cognition **16**, 709-730. (10.1017/S1366728912000727)

[8] Nichols J. 2003 Diversity and stability in language. In *Handbook of historical linguistics* (eds RD Janda, BD Joseph), pp. 283–310. London, UK: Blackwell.

[9] Van Gijn R, Wahlström M. In press. Linguistic areas. In *Language contact: bridging the gap between individual interactions and areal patterns* (eds R van Gijn, M Wahlström, H Ruch, A Hasse).

[10] Heine B, Kuteva T. 2006 The changing languages of Europe. Oxford, UK: Oxford University Press.

[11] Masica C. 2001 The definition and significance of linguistic areas: methods, pitfalls, and possibilities (with special reference to the validity of South Asia as a linguistic area). In *Tokyo Symposium on South Asian languages: contact, convergence, and typology* (eds P Bhaskararao, KV Subbarao), pp. 205–267. New Delhi, India: Sage Publications.

[12] Stolz T. 2006 All or nothing. In *Linguistic areas: convergence in historical and typological perspective* (eds Y Matras, A McMahon, N. Vincent), pp. 32–50. Basingstoke, UK: Palgrave MacMillan.

[13] Van Gijn R. 2020 Separating layers of information: the anatomy of contact zones. In *My workplace that is my head: a Festschrift for Pieter Muysken* (eds Nl Smith, T Veenstra, E Aboh), pp. 161–178. Amsterdam, The Netherlands: John Benjamins.

[14] Croft W. 2008 Evolutionary linguistics. Annu. Rev. Anthropol. **37**, 219-234. (10.1146/annurev.anthro.37.081407.085156)

[15] Gray RD, Greenhill SJ, Ross RM. 2007 The pleasures and perils of darwinizing culture (with phylogenies). Biol. Theory **2**, 360-375. (10.1162/biot.2007.2.4.360)

[16] Bickel B. 2015 Distributional typology: statistical inquiries into the dynamics of linguistic diversity. In *The Oxford handbook of linguistic analysis* (eds B Heine, H Narrog), 2nd edn, pp. 901–923. Oxford, UK: Oxford University Press.

[17] Croft W. 2003 Typology and universals, 2nd edn. Cambridge, UK: Cambridge University Press.

[18] Gibson E, Futrell R, Piantadosi SP, Dautriche I, Mahowald K, Bergen L, Levy R. 2019 How efficiency shapes human language. Trends Cogn. Sci. **23**, 389-407. (10.1016/j.tics.2019.02.003) 31006626

[19] Kirby S. 2017 Culture and biology in the origins of linguistic structure. Psychon. Bull. Rev. **24**, 118-137. (10.3758/s13423-016-1166-7) 28120320 PMC5325872

[20] MacDonald MC. 2013 How language production shapes language form and comprehension. Front. Psychol. **4**, 226. (10.3389/fpsyg.2013.00226) 23637689 PMC3636467

[21] Dryer MS. 2013 Polar questions. In *The world atlas of language structures online* (eds MS Dryer, M Haspelmath). Leipzig, Germany: Max Planck Institute for Evolutionary Anthropology.

[22] Boas F. 1911 Handbook of American Indian languages, vol. 1. Washington, DC: Smithsononian Institution, Bureau of American Ethnology.

[23] Boduen-de Kurtenè IA, směšannom harakterě vsěh jazykov O. 1901 Žurnal Ministerstva narodnago prosvěščenija **337**, 12-24.

[24] Sandfeld K. 1926 Balkanfilologien: en oversigt over dens resultater og problemer. Copenhagen, Denmark: Københavns Universitet.

[25] Trubetzkoy N. 1923 Vavilonskaja bašnja i smešenie jazykov. Evrazijskij vremennik **3**, 107-124.

[26] Daumé III H. 2009 Non-parametric Bayesian areal linguistics. (http://arxiv.org/abs/0906.5114)

[27] Towner M, Grote M, Venti J, Mulder M. 2012 Cultural macroevolution on neighbor graphs: vertical and horizontal transmission among western North American Indian societies. Hum. Nat. (Hawthorne, N.Y.) **23**, 283-305. (10.1007/s12110-012-9142-z) 22791406

[28] Murawaki Y. 2020 Latent geographical factors for analyzing the evolution of dialects in contact. In *Proc. 2020 Conf. on Empirical Methods in Natural Language Processing (EMNLP)*, pp. 959–976.

[29] Michael L, Chang W, Stark T. 2014 Exploring phonological areality in the circum-Andean region using a naive Bayes classifier. Lang. Dyn. Change 4, 27-86. (10.1163/22105832-00401004)

[30] Chang W, Michael L. 2014 A relaxed admixture model of language contact. Lang. Dyn. Change **4**, 1-26. (10.1163/22105832-00401005)

[31] Dryer MS, Haspelmath M (eds). 2013 The world atlas of language structures online. Leipzig, Germany: Max Planck Institute for Evolutionary Anthropology.

[32] Dryer MS. 2013 Order of subject, object and verb. In *The world atlas of language structures online* (eds MS Dryer, M Haspelmath). Leipzig, Germany: Max Planck Institute for Evolutionary Anthropology.

[33] Napoli DJ, Sutton-Spence R. 2014 Order of the major constituents in sign languages: implications for all language. Front. Psychol. **5**, 376. (10.3389/fpsyg.2014.00376) 24860523 PMC4026690

[34] Spiegelhalter DJ, Best NG, Carlin BP, Van Der Linde A. 2002 Bayesian measures of model complexity and fit. J. R. Stat. Soc. Ser. B **64**, 583-639. (10.1111/1467-9868.00353)

[35] Gao H, Bryc K, Bustamante CD. 2011 On identifying the optimal number of population clusters via the deviance information criterion. PLoS ONE **6**, e21014. (10.1371/journal.pone.0021014) 21738600 PMC3125185

[36] Watanabe S, Opper M. 2010 Asymptotic equivalence of Bayes cross validation and widely applicable information criterion in singular learning theory. J. Mach. Learn. Res. **11**, 3571-3594.

[37] Vehtari A, Gelman A, Gabry J. 2017 Practical Bayesian model evaluation using leave-one-out cross-validation and WAIC. Stat. Comput. **27**, 1413-1432. (10.1007/s11222-016-9696-4)

[38] Rambaut A, Drummond AJ, Xie D, Baele G, Suchard MA. 2018 Posterior summarization in Bayesian phylogenetics using Tracer 1.7. Syst. Biol. **67**, 901. (10.1093/sysbio/syy032) 29718447 PMC6101584

[39] Büttner TT. (1983). Las lenguas de los Andes centrales: estudios sobre la clasificación genética, areal y tipológica. *Madrid, Spain: Cultura Hispánica del Instituto de cooperación iberoamericana.*.

[40] Derbyshire DD, Pullum GK. 1986 Introduction. In *Handbook of Amazonian languages 1* (eds DC Derbyshire, GK Pullum), pp. 1–28. Berlin, Germany: Mouton de Gruyter.

[41] Dixon RMW, Aikhevald AY. 1999 Introduction. In *The Amazonian languages* (eds RMW Dixon, AY Aikhenvald), pp. 1–21. Cambridge, UK: Cambridge University Press.

[42] Torero Fernández de Cordoba A. 2002 Idiomas de los Andes: Lingüística e Historia. Lima, Peru: Editorial Horizonte.

[43] Epps P, Michael L. 2017 *The areal linguistics of Amazonia*, pp. 934–963. Cambridge Handbooks in Language and Linguistics. Cambridge, UK: Cambridge University Press.

[44] Urban M. 2019 Is there a central andean linguistic area? A view from the perspective of the ‘minor’ languages. J. Language Contact **12**, 271-304. (10.1163/19552629-01202002)

[45] Matula DW, Sokal RR. 1980 Properties of Gabriel graphs relevant to geographic variation research and the clustering of points in the plane. Geogr. Anal. **12**, 205-222. (10.1111/j.1538-4632.1980.tb00031.x)

[46] Adelaar WFH. 2004 The languages of the Andes. Cambridge, UK: Cambridge University Press.

[47] Constenla Umaña A. 1991 Las lenguas del área intermedia: introducción a su estudio areal, 1st edn. San José, Costa Rica: Universidad de Costa Rica.

[48] Curnow TJ. 1998 Why Paez is not a Barbacoan language: the nonexistence of ‘moguex’ and the use of early sources. Int. J. Am. Linguist. **64**, 338-351. (10.1086/466365)

[49] Kohlberger M. 2020 A grammatical description of Shiwiar. PhD thesis, Rijksuniversiteit te Leiden.

[50] Valenzuela P. 2015 Qué tan ‘amazónicas’ son las lenguas kawapana? Contacto con las lenguas centro-andinas y elementos para un área lingüística intermedia. Lexis **39**, 5-56.

[51] Wise MR. 2014 Rastros desconcertantes de contactos entre idiomas y culturas a lo largo de los contrafuertes orientales de los Andes del Perú. In *Estudios sobre lenguas Andinas y Amazónicas: Homenaje a Rodolfo Cerrón-Palomino* (eds WFH Adelaar, P Valenzuela, R Zariquiey), pp. 305–326. Lima, Peru: Fondo Editorial, Universidad Católica del Perú.

[52] Adelaar WFH. 2012 Languages of the Middle Andes in areal-typological perspective: emphasis on Quechuan and Aymaran. In *The indigenous languages of South America. A comprehensive guide* (eds L Campbell, V Grondona), pp. 575–624. Berlin, Germany: De Gruyter.

[53] Wise MR. 2011 Raostros desconcertantes de contactos entre idiomas y culturas a lo largo de los contrafuertes orientales de los andes del Perú. In *Estudios sobre lenguas andinas y amazónicas. Homenaje a Rodolfo Cerrón-Palomino* (eds W Adelaar, P Valenzuela, R Zariquiey), pp. 305–326. Lima, Peru: Fondo Editorial de la Pontificia Universidad Católica del Perú.

[54] Eriksen L. 2011 Nature and culture in prehistoric Amazonia: using G.I.S. to reconstruct ancient ethnogenetic processes from archaeology, linguistics, geography, and ethnohistory. PhD thesis, Lund University, Lund.

[55] Jolkesky MPDV. 2016 Estudo arqueo-ecolinguístico das terras tropicais sul-americanas. PhD thesis, Universidade de Brasília.

[56] Krasnoukhova O. 2012 The noun phrase in the languages of South America. PhD thesis, Radboud Universiteit Nijmegen.

[57] Krasnoukhova O. 2014 Argument marking patterns in South American languages. PhD thesis, Radboud Universiteit Nijmegen.

[58] van Gijn R. 2014 The Andean foothills and adjacent Amazonian fringe. In *The native languages of South America. Origins, development, typology* (eds L O’Connor, P Muysken), pp. 102–125. Cambridge, UK: Cambridge University Press.

[59] van Gijn R, Muysken P. 2020 Highland-lowland relations: a linguistic view. In *Rethinking the Andes-Amazonia ’Divide’. A cross-disciplinary exploration* (eds AJ Pearce, DG Beresford-Jones, P Heggarty), pp. 178–210. London, UK: University College Press.

[60] Crevels M, van der Voort H. 2008 The Guaporé-Mamoré region as a linguistic area. In *From linguistic areas to areal linguistics* (ed. P Muysken). Studies in Language Companion Series, vol. 90, pp. 151–179. Amsterdam, The Netherlands: John Benjamins.

[61] Kopitar J. 1945[1829] Albanische, walachische und bulgarische sprache. In *Jerneja Kopitarja spisov. II. del* (ed. R Nahtigal), pp. 227–273. Akademija znanosti i umetnosti.

[62] Friedman VA, Joseph BD. 2017 Reassessing sprachbunds. In *The Cambridge handbook of areal linguistics* (ed. R Hickey), pp. 55–87. Cambridge, UK: Cambridge University Press.

[63] Joseph B. 2010 Language contact in the Balkans. In *The handbook of language contact* (ed. R Hickey), pp. 618–633. Wiley-Blackwell.

[64] Friedman VA. 2006 Balkans as a linguistic area. In *Encyclopedia of language & linguistics* (ed. K Brown), vol. 1, 2nd edn, pp. 657–672. Oxford, UK: Elsevier.

[65] Goła̧b Z. 1997 The ethnic background and internal linguistic mechanism of the so-called Balkanization of Macedonian. Balkanistica **10**, 13-19.

[66] Lindstedt J. 2000 Linguistic Balkanization: contact-induced change by mutual reinforcement. Stud. Slavic General Linguist. **28**, 231-246.

[67] Alexander R. 2000 Tracking Sprachbund boundaries: word order in the Balkans. Stud. Slavic General Linguistics **28**, 9-27.

[68] Ivć P. 1969 *Balkan Slavic migrations in the light of South Slavic dialectology*, pp. 66–86. The Hague, The Netherlands: De Gruyter Mouton.

[69] Bickel B, Nichols J. 2006 Oceania, the Pacific Rim, and the theory of linguistic areas. Proc. Berkeley Linguistics Soc. **32**, 3-15. (10.3765/bls.v32i2.3488)

[70] Cathcart CA. 2020 A probabilistic assessment of the Indo-Aryan inner–outer hypothesis. J. Hist. Linguist. **10**, 42-86. (10.1075/jhl.18038.cat)

[71] Syrjänen K, Honkola T, Lehtinen J, Leino A, Vesakoski O. 2016 Applying population genetic approaches within languages. Lang. Dyn. Change **6**, 235-283. (10.1163/22105832-00602002)

[72] Reesink G, Singer R, Dunn M. 2009 Explaining the linguistic diversity of Sahul using population models. PLoS Biol. **7**, e1000241. (10.1371/journal.pbio.1000241) 19918360 PMC2770058

[73] Felsenstein J. 1973 Maximum likelihood and minimum-steps methods for estimating evolutionary trees from data on discrete characters. Syst. Biol. **22**, 240-249. (10.1093/sysbio/22.3.240)

[74] Everett C, Blasi DE, Roberts SG. 2015 Climate, vocal folds, and tonal languages: connecting the physiological and geographic dots. Proc. Natl Acad. Sci. USA **112**, 1322-1327. (10.1073/pnas.1417413112) 25605876 PMC4321236

[75] Everett C. 2013 Evidence for direct geographic influences on linguistic sounds: the case of ejectives. PLoS ONE **8**, e65275. (10.1371/journal.pone.0065275) 23776463 PMC3680446

[76] Dediu D, Ladd RD. 2007 Linguistic tone is related to the population frequency of the adaptive haplogroups of two brain size genes, *ASPM* and *Microcephalin*. Proc. Natl. Acad. Sci. USA **104**, 10 944-10 949. (10.1073/pnas.0610848104) PMC1904158 17537923

[77] Blasi DE, Moran S, Moisik SR, Widmer P, Dediu D, Bickel B. 2019 Human sound systems are shaped by post-Neolithic changes in bite configuration. Science **363**, eaav3218. (10.1126/science.aav3218) 30872490

[78] Hay J, Bauer L. 2007 Phoneme inventory size and population size. Language **83**, 388-400. (10.1353/lan.2007.0071)

[79] Xie W, Lewis PO, Fan Y, Kuo L, Chen M-H. 2011 Improving marginal likelihood estimation for Bayesian phylogenetic model selection. Syst. Biol. **60**, 150-160. (10.1093/sysbio/syq085) 21187451 PMC3038348

[80] Gelman A, Carlin JB, Stern HS, Dunson DB, Vehtari A, Rubin DB. 2013 Bayesian data analysis. New York, NY: CRC Press.

[81] Delaunay B. 1934 Sur la sphere vide. Izv. Akad. Nauk SSSR, Otdelenie Matematicheskii i Estestvennyka Nauk **7**, 1-2.

[82] Lyman R, O’Brien M. 2003 Cultural traits: units of analysis in early twentieth-century anthropology. J. Anthropol. Res. **59**, 225-250. (10.1086/jar.59.2.3631642)

[83] Kirby K*et al.* 2016 D-PLACE: a global database of cultural, linguistic and environmental diversity. PLoS ONE **11**, e0158391. (10.1371/journal.pone.0158391) 27391016 PMC4938595

[84] Nunn CL. 2011 The comparative approach in evolutionary anthropology and biology. Chicago, IL: University of Chicago Press.

[85] Richerson PJ, Boyd R. 2008 Cultural evolution: accomplishments and future prospects, pp. 75-99. Seattle, WA: University of Washington Press.

[86] Moravec J, Atkinson QD, Bowern C, Greenhill SJ, Jordan F, Ross R, Gray RD, Marsland S, Cox M. 2018 Post-marital residence patterns show lineage-specific evolution. Evol. Hum. Behav. **39**, 594-601. (10.1016/j.evolhumbehav.2018.06.002)

[87] Turchin P*et al.* 2018 Quantitative historical analysis uncovers a single dimension of complexity that structures global variation in human social organization. Proc. Natl Acad. Sci. USA **115**, E144-E151. (10.1073/pnas.1708800115) 29269395 PMC5777031

[88] Watts J, Sheehan O, Atkinson QD, Bulbulia J, Gray RD. 2016 Ritual human sacrifice promoted and sustained the evolution of stratified societies. Nature **532**, 228-231. (10.1038/nature17159) 27042932

